# Transcutaneous electrical nerve stimulation enhances locomotor adaptation savings in people with multiple sclerosis

**DOI:** 10.1093/braincomms/fcaf255

**Published:** 2025-06-25

**Authors:** Andrew C Hagen, Tyler T Whittier, Jaclyn A Stephens, Brett W Fling

**Affiliations:** Department of Health and Exercise Science, Colorado State University, Fort Collins, CO 80523, USA; Department of Food Systems, Nutrition and Kinesiology, Montana State University, Bozeman, MT 59717, USA; Department of Health and Exercise Science, Colorado State University, Fort Collins, CO 80523, USA; Molecular, Cellular and Integrative Neurosciences Program, Colorado State University, Fort Collins, CO 80523, USA; Department of Health and Exercise Science, Colorado State University, Fort Collins, CO 80523, USA; Molecular, Cellular and Integrative Neurosciences Program, Colorado State University, Fort Collins, CO 80523, USA

**Keywords:** multiple sclerosis, locomotor adaptation, transcutaneous electrical nerve stimulation, functional near-infrared spectroscopy, motor learning

## Abstract

Locomotor adaptation on a split-belt treadmill can improve gait symmetry across various clinical populations, including people with multiple sclerosis (PwMS). As many PwMS experience sensory impairments, mobility interventions relying on sensory prediction errors may be less effective. Transcutaneous electrical nerve stimulation (TENS) has been shown to amplify sensorimotor function in PwMS and healthy controls, but its influence on motor learning remains unexplored. This randomized crossover trial investigated the effects of TENS on locomotor adaptation and cortical activation in PwMS. In total, 28 PwMS and 20 age- and sex-matched healthy controls completed two locomotor adaptation sessions, one with active TENS and one with inactive TENS. Locomotor adaptation was evaluated using step length asymmetry, quantified across four outcome metrics: adaptation magnitude, early change, after-effect and savings. Functional near-infrared spectroscopy recorded cortical activation, and linear mixed-effect models assessed group, visit and TENS condition effects on behavioural and cortical activation outcomes. PwMS exhibited reduced adaptation magnitude compared with healthy controls. TENS did not influence early change (representing adaptation rate) but significantly improved adaptation savings for PwMS who received TENS during their second visit only (initial savings: adj-*P* = 0.005, *d* = 1.35; early savings: adj-*P* = 0.014, *d* = 1.13). Additionally, both PwMS and healthy controls exhibited decreased cortical activation during locomotor adaptation with TENS, particularly in the dorsal premotor cortex for PwMS (adj-*P* = 0.019, *d* = 0.84). These findings indicate that TENS promotes the retention of prior locomotor adaptation, enhancing the efficiency of relearning. Additionally, reduced cortical activation with TENS in both groups indicates reduced cortical reliance during adaptation. Together, these effects suggest that TENS could have broader utility for enhancing motor learning in populations with sensory impairments, potentially leading to amplified retention and automaticity during motor rehabilitation paradigms.

## Teaser

Hagen *et al*. investigated whether transcutaneous electrical nerve stimulation (TENS) could enhance motor learning in response to a walking perturbation in people with multiple sclerosis. TENS led to greater motor learning savings and reduced cortical activation during learning, suggesting that TENS may improve learning retention and decrease cortical demands.

## Graphical Abstract

**Graphical Abstract:**
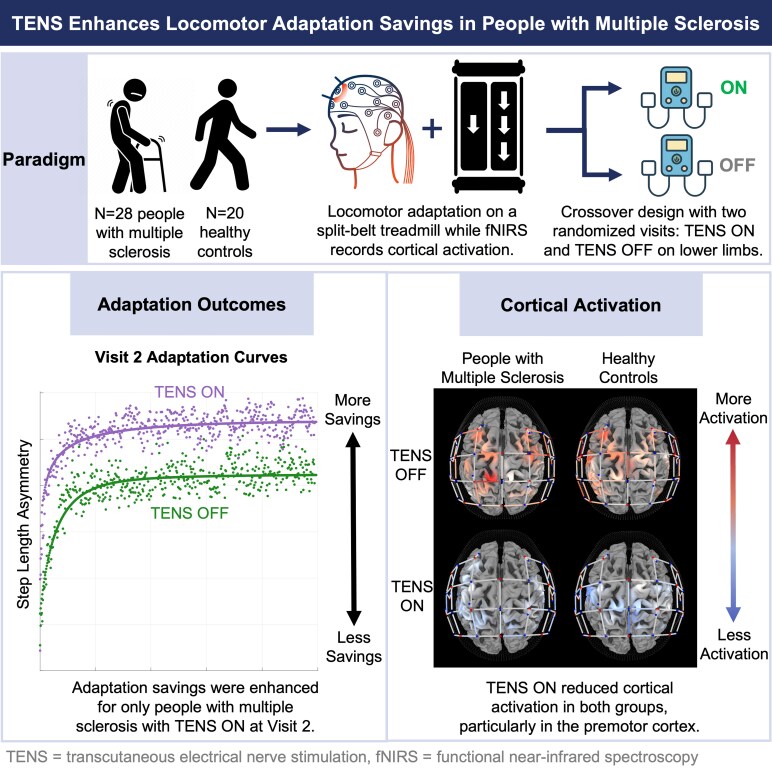

## Introduction

Many of the earliest symptoms of multiple sclerosis are sensory in nature, including numbness, tingling, restless limb and pain,^1,2^ affecting around 85% of people with multiple sclerosis (PwMS) in their first year of diagnosis.^3^ Importantly, these sensory symptoms are likely the main driver of poor motor performance seen in PwMS.^4-6^ Split-belt treadmill walking, where the speed of each limb is controlled independently, is a form of sensorimotor adaptation that can improve spatial, temporal and kinetic gait symmetry in many populations,^7,8^ including PwMS.^9,10^ This locomotor adaptation is highly dependent on sensory input and cerebellar function.^11,12^ Given the sensory impairments and cerebellar damage in PwMS,^13-15^ even slight sensory disruptions could significantly impact adaptation, as the cerebellum relies on the fastest afferent fibres to support timely and accurate prediction.^16^ Communication between the cortex and cerebellum is also imperative for locomotor adaptation, with research demonstrating that cerebellar-brain inhibition, or the cerebellum's inhibitory influence on the motor cortex, is proportional to adaptation.^17^ Notably, other evidence indicates that, unlike controls, postural adaptation in PwMS is not associated with cortico-cerebellar connectivity strength, implying PwMS may use compensatory strategies, such and increased cortical activation to support motor learning.^18,19^ Accordingly, there is a critical need to develop therapeutic strategies that enhance sensory function and support precise motor control, addressing significant gaps in both research and clinical practice.

Transcutaneous electrical nerve stimulation (TENS) has been demonstrated to improve sensorimotor integration in healthy and clinical populations, in skilled motor tasks including postural balance,^20^ tactile perception,^21,22^ manual dexterity,^23,24^ and walking.^25-27^ However, very few studies have investigated the effect of TENS on motor learning.^28,29^ TENS is hypothesized to preferentially recruit Aα and Aβ afferent fibres,^20,30,31^ thereby increasing afferent excitability. Although direct measurements of axonal excitability are limited, TENS has been shown to increase H-reflex amplitude and cortical excitability,^20,32-34^ suggesting enhanced afferent signalling. These effects have been suggested to reduce sensorimotor uncertainty by improving the accuracy of state estimation during movement.^28^ Separately, TENS alters activation patterns in sensorimotor cortical regions,^33,35^ indicating broader effects on motor control.

While most research has focused on the cerebellum,^12,36,37^ the influence of cortical regions during locomotor adaptation remains less clear.^38-40^ Neuroimaging studies have shown that PwMS exhibit greater sensorimotor cortex activation and altered functional connectivity compared with controls during upper and lower extremity motor tasks, likely as a compensatory mechanism for impaired sensory and cerebellar function.^41,42^ Building on these observations, the present study aimed to investigate the effect of TENS on locomotor adaptation in PwMS. We hypothesized that TENS would enhance locomotor adaptation, specifically by increasing the rate of stepping symmetry improvements. Additionally, we hypothesized reduced activation in sensorimotor cortical regions, as measured with functional near-infrared spectroscopy (fNIRS), when TENS was applied, indicating less cortical involvement during locomotor adaptation.

## Materials and methods

### Participants

In this randomized controlled trial (NCT05878873), eligible participants were aged 18–75 years, and either had a diagnosis of relapsing–remitting multiple sclerosis or were a neurotypical healthy control (HC). PwMS were required to have an Expanded Disability Status Scale (EDSS) score below five and not be in an active relapse. Exclusion criteria included inability to walk 500 m unassisted, a lower limb injury or surgery within 6 months, use of medications that impair balance, history of another neural or balance impairment (e.g. traumatic brain injury, stroke and vestibular disease) or current pregnancy. This study adhered to the Declaration of Helsinki and was approved by the Colorado State University Biomedical Institutional Review Board (protocol code: 4795). A preliminary analysis of five PwMS revealed a large effect (*d* = 1.01) for change in fNIRS-measured activation within the premotor cortex from Baseline to Early Adapt. Based on this effect, a power analysis indicated at least 10 participants per group were needed to maintain an alpha of 0.05 and a power of 0.8. However, to account for smaller effects in other regions, sample size was increased to 15 per group for PwMS. For HCs, 10 participants per group was deemed sufficient based on previous locomotor adaptation studies.^43^

Demographics and disease characteristics were collected after screening and informed consent. Measures included the EDSS, 12-Item Multiple Sclerosis Walking Scale, Modified Fatigue Impact Scale, Beck Depression Inventory, oral Symbol Digit Modalities Test, hallux vibration perception threshold with a Rydel-Seiffer tuning fork, rate of perceived exertion and Modified Clinical Test of Sensory Integration in Balance (mCTSIB). For the mCTSIB, primary measures included sway during the proprioceptive condition (eyes closed on firm surface) and the composite sway score across all conditions.

### Study design

**Figure 1:**
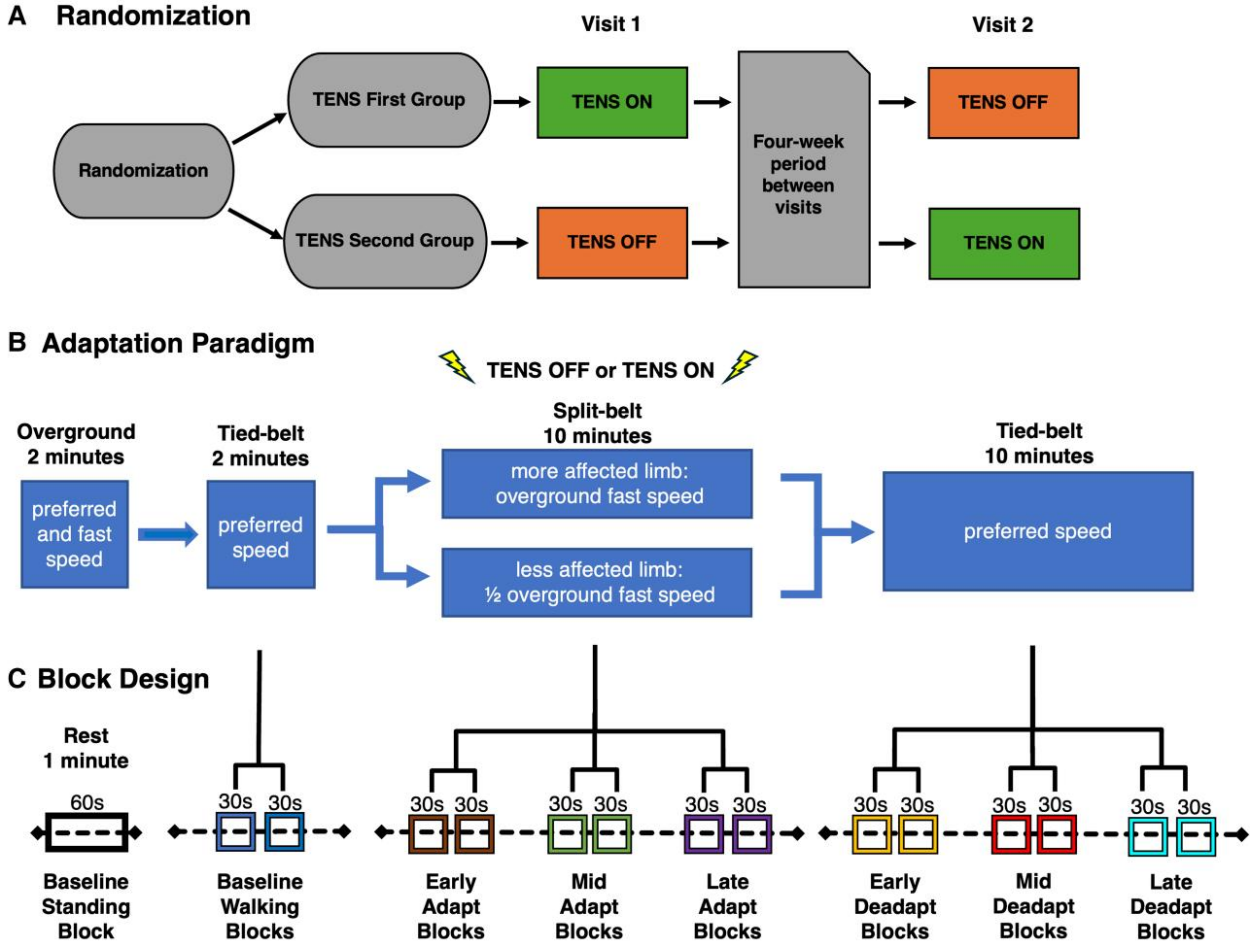
**Study design.** (**A**) Crossover design and randomization of transcutaneous electrical nerve stimulation (TENS) for each visit. The first group received TENS ON during Visit 1 and TENS OFF during Visit 2, while the second group had the reverse order. A 4-week washout period separated the visits. (**B**) Locomotor adaptation paradigm. Participants first completed baseline overground walking at preferred and fast speeds, followed by tied-belt treadmill walking at their preferred speed. During the split-belt adaptation phase (10 min), the more affected limb was set to the overground fast speed, while the less affected limb was set to half the overground fast speed. TENS was active during adaptation phases only. The protocol concluded with tied-belt treadmill walking (10 min) at the preferred speed. (**C**) Functional near-infrared spectroscopy (fNIRS) imaging block design. Following a 60-s standing rest period, pairs of 30-s blocks were averaged to quantify cortical activation for each treadmill walking timepoint in the baseline, adaptation and deadaptation phases.

In this crossover design, PwMS and HC participants were pseudo-randomized to begin in either the TENS First or TENS Second condition. Randomization was performed using a random number generator and was counterbalanced across age, sex and fast limb to ensure group balance. A predefined list of participant numbers with corresponding randomized assignments was generated prior to enrolment. Participants were enrolled sequentially and assigned to a group based on this list. The TENS first group received active TENS (TENS ON) during the first locomotor adaptation visit and inactive TENS (TENS OFF) during the second visit while the TENS second group received these conditions in the reverse order, with a 4-week interval between visits (Fig. 1A). Importantly, TENS was active only during the adaptation phase of the paradigm, when the treadmill belts moved at different speeds.

Overground walking assessments were conducted in a 30-m hallway during Visit 1 to determine treadmill speeds. Participants performed two separate 2-min walk trials: one at their preferred speed and one at their fastest comfortable speed they could maintain for 15 min, while turning at each end of the hallway as needed. The ‘fast’ belt on the split-belt treadmill during locomotor adaptation was set to this fast walk speed while the ‘slow’ belt was set to half of this fast walk speed (2:1 ratio). The ‘fast’ limb was the more affected limb in PwMS, confirmed as the shorter overground step length. In HCs, fast limb assignments were counterbalanced to match the distribution observed in PwMS and were aligned by age and sex. During treadmill walking, participants completed a 2-min walk with the belts tied at their preferred speed, followed by a 10-min adaptation phase with the belts split and a 10-min deadaptation phase with the belts tied again at their preferred speed (Fig. 1B). Participants were secured in a harness without bodyweight support, instructed to hold the handrails and fixed their gaze on a crosshair target positioned ahead on a large backdrop.

### TENS parameters

During all visits, participants had TENS electrodes placed over the muscle bellies of the bilateral tibialis anterior and rectus femoris, while the TENS units (LG–TECELITE, LGMedSupply) were mounted to the NIRSport2 device. These locations were selected to target muscles involved in dorsiflexion, hip flexion and knee extension, common areas of impairment that affect walking in PwMS.^44,45^ Furthermore, previous studies have successfully improved gait performance in PwMS using these electrode locations.^28,46,47^ Stimulation consisted of biphasic bursts (seven 0.15 ms pulses) at 5 Hz through 2 × 4 inch electrode pairs, based on recent evidence suggesting that bursting TENS is most effective during walking.^47^ To reduce electrical impedance, the skin over the muscles was shaved to prior to pad placement. Electrode placement and stimulation parameters (e.g. pulse width, frequency) were identical for both PwMS and HC groups. TENS amplitude was individualized for each participant based on their motor threshold. Stimulation intensity was increased in 1 mA increments for each muscle until non-voluntary contractions were observed by the participant or investigator. Contractions were defined as visible muscle twitching or movement at the joint or a palpable muscle response during light manual contact by the investigator. Once threshold was identified, amplitude was set to 2 mA below this threshold for each muscle.^28^

### Gait data analysis

During overground walking, gait parameters were measured using six APDM opal inertial sensors and processed using APDM Mobility Lab (v. 2.0, APDM Wearable Technologies). During treadmill walking, participants were outfitted with 16 retroreflective markers placed according to the Vicon Plug in Gait Lower Body model, while 10 Vicon T010 infrared cameras surrounding the treadmill captured motion at 100 Hz. This instrumented treadmill (Model 4060-10, Bertec Corp) recorded ground reaction forces at 1000 Hz, and had two motors that allowed independent control of each belt. Force data were filtered using a fourth-order zero-lag Butterworth filter (300 Hz low-pass cutoff) and marker positions were filtered using a fifth-order spline-interpolating Woltring filter in Vicon Nexus (v. 2.15, Vicon Motion Systems). For this study, the primary measure during treadmill walking was step length asymmetry (SLA), which has been shown to adapt robustly during split-belt treadmill walking.^48^ Step length was calculated as the anterior–posterior distance in mm between heel markers at heel strike for each limb. A body-centered model of heel location was used to account for participant translation within strides,^49^ and SLA was normalized to participant stride length using the following equation:


(1) $$\mathrm{SLA}(i)=\frac{\;\mathrm{step}\;\mathrm{lengt}h_{\mathrm{fast}\;\mathrm{limb}}(i)\;(\mathrm{mm})-\mathrm{step}\;\mathrm{lengt}h_{\mathrm{slow}\;\mathrm{limb}}(i)\;(\mathrm{mm})}{\;\mathrm{step}\;\mathrm{lengt}h_{\mathrm{fast}\;\mathrm{limb}}(i)\;(\mathrm{mm})+\mathrm{step}\;\mathrm{lengt}h_{\mathrm{slow}\;\mathrm{limb}}(i)\;(\mathrm{mm})}$$

To improve comparability across participants, the SLA curves for adaptation and deadaptation were baseline-adjusted by subtracting each participant's visit-specific average SLA at Baseline (last 30 strides) from their SLA at each stride. Baseline adjustment removed individual and visit-specific offsets, ensuring that differences in SLA curves reflect changes in adaptation rather than baseline variability.^50^ Following, baseline-adjusted adaptation and deadaptation SLA curves were fitted to a single exponential model (*y* = *a* × *e*^*bn*^ + *c*) using the algorithm and parameters established by Rashid *et al*.^51^ to more accurately quantify the underlying learning process. Curve fitting was performed on each participant's stride-by-stride data using a particle swarm optimization algorithm to minimize squared error. Although locomotor adaptation is often represented by a two-rate model, the single exponential provided a more consistent fit across our PwMS sample without requiring individualized parameter adjustments.^52^ From these exponential models, average SLA was calculated at six timepoints per visit: Initial Adapt (Strides 1–5), Early Adapt (Strides 6–30), Late Adapt (last 30 strides), Initial Deadapt (Strides 1–5), Early Deadapt (Strides 6–30) and Late Deadapt (last 30 strides). Subsequently, these averages were used to calculate the following outcomes to characterize locomotor adaptation^53^: (i) early change, defined as the average SLA during the Early Adapt and Early Deadapt phases; (ii) adaptation magnitude, calculated as the difference in SLA between the Early and Late phases of Adapt and Deadapt; (iii) after-effect, defined as the difference between Early Deadapt and Baseline SLA; and (iv) savings, quantified as the difference in SLA from Visit 1 to Visit 2 at Initial and Early Adapt, reflecting the rate of relearning.

### fNIRS acquisition

During treadmill walking, participants had a NIRSport2 (NIRx Medical Technologies) mobile fNIRS device attached to their back, which acquired and wirelessly transmitted data to Aurora software (v. 2021.9, NIRx Medical Technologies) at a 6.1 Hz sampling rate. A block design with two 30-s blocks per timepoint was used to quantify cortical activation, and data were averaged across each block pair (Fig. 1C). The cap montage was designed using the fNIRS Optodes Location Decider toolbox^54^ in MATLAB (v. R2022b) and its Brodmann atlas to create source-detector pairs (i.e. channels) over premotor, sensorimotor and posterior parietal regions of interest (ROIs), as these areas are implicated in gait modulation.^55^ This montage contained 16 LED source optodes (760 and 850 nm) and 15 detector optodes creating 48 channels that captured changes in oxyhaemoglobin (HbO), deoxyhaemoglobin (HbR) and total haemoglobin. Eight short-distance detectors measured scalp perfusion, or blood flow to the scalp, as opposed to blood flow in the cortex, and were used during preprocessing to regress out confounding signals including motion, heart rate and blood pressure.^56^ Before collection, signal optimization (i.e. source brightness calibration) and cap preparation steps (e.g. moving hair, adjusting optode tension) were repeated until all channels reached acceptable levels of signal quality (>0.5 mV).

### fNIRS preprocessing and analysis

Raw fNIRS data were processed using Satori (v. 1.8, NIRx Medical Technologies). Initial preprocessing steps of conversion and spatial registration are performed automatically in this software. Raw light intensity data were converted to optical density values and then to concentrations of HbO, HbR and total haemoglobin using the Modified Beer–Lambert law. The outcomes of this study are based on HbO values, as HbO is the most reported chromophore in fNIRS research and provides the most direct measure of cortical activation.^57^ HbO reflects oxygen delivery to active brain regions and is most sensitive to the haemodynamic response. HbR reflects oxygen extracted by the tissue typically showing inverse changes to HbO. HbR results are available in Supplementary Fig. 1. Since the primary outcomes were relative beta weights and the cap montage used a consistent source-detector separation (35–40 mm) across all channels, a differential pathlength factor was not applied, as this minimized pathlength variability and made it unnecessary for comparing relative activation between conditions.^58^ Following, individual channel data were spatially registered to the montage and displayed for visual inspection and confirmation of signal quality. Events were manually created for each participant using stimulus trigger markers for each block in the block design.

Subsequently, motion artefact correction and spike removal were performed using the default parameters in Satori. Spikes, or brief, high-amplitude fluctuations typically caused by motion were identified using 10 iterations, 5-s lag, 3.5 threshold and 0.5 influence. Temporal Derivative Distribution Repair was then applied to restore high-frequency bands with monotonic interpolation. Next, short-channel regression was performed for each channel using the highest correlated short-distance detector. Temporal filtering consisted of the default Satori parameters including a high-pass Butterworth filter (0.01 Hz) and low-pass Gaussian smoothing (0.4 Hz). Additionally, Z-transform normalization was performed to make data more comparable between participants. The only default preprocessing step omitted was automatic channel rejection based on scalp coupling index. However, scalp coupling indexes were calculated for each channel to identify potential outliers post-processing.

Following standardized preprocessing, HbO beta weights were calculated using a general linear model to quantify haemodynamic response strength (double-gamma function model) at each timepoint. These betas isolate task-related activation magnitude between conditions, making them robust to baseline fluctuations and noise. To quantify channel-wide activation, HbO betas were averaged across all channels in the array. Additionally, HbO betas from each channel were organized into different ROI clusters.^59,60^ Channels included in each ROI were determined *a priori* based on the highest Brodmann area (BA) specificity, while avoiding channels that overlap multiple ROIs and ensuring symmetrical bilateral correspondence. These bilateral clusters included the dorsal and ventral premotor cortex (PMd, PMv, BA6), primary motor cortex (M1, BA4), primary somatosensory cortex (S1, BA3,1,2), superior parietal lobule (SPL, BA5,7) and inferior parietal lobule (IPL, BA39,40) (Supplementary Table 1). Contrasts were then created between the averaged Baseline and Early Adapt blocks to isolate activation specific to adaptation alone, and independent of treadmill walking effects.

### Statistical analysis

Linear mixed-effect models were used to identify differences in adaptation and fNIRS outcomes, with group (PwMS versus HC), visit (Visit 1 versus Visit 2) and TENS condition (TENS ON versus TENS OFF), along with their interactions, as fixed effects, and participant number as a random effect. Assumptions of linearity, normality of residuals and homoscedasticity were confirmed for each model. Following model creation, a repeated-measure ANOVA was performed for each adaptation outcome (adaptation magnitude, early change, after-effect and savings) and for HbO beta, both at the channel-wide and ROI levels. Type III ANOVAs with Satterthwaite's method for degrees of freedom estimation were used for all analyses. *Post hoc* comparisons were conducted using estimated marginal means (EMM) to further investigate significant main effects and interactions. *P*-values were corrected using the Benjamini–Hochberg false discovery rate within each outcome and group (e.g. ROI *P*-values within the PwMS group were corrected for across all other ROIs) when applicable. Cohen's *d* was calculated to quantify effect sizes. Backward stepwise regression models were conducted to assess the influence of demographic covariates on primary outcome models. Pearson correlations between significant changes in HbO beta and savings were computed to explore their relationship. Finally, linear regression models and backward stepwise regression models were conducted to assess the predictability of HbO beta for savings. All statistical analyses were conducted using the lme4 and emmeans packages within R (v. 4.4.0) and were two-tailed with an alpha threshold set at 0.05.

## Results

### Participants

**Figure 2:**
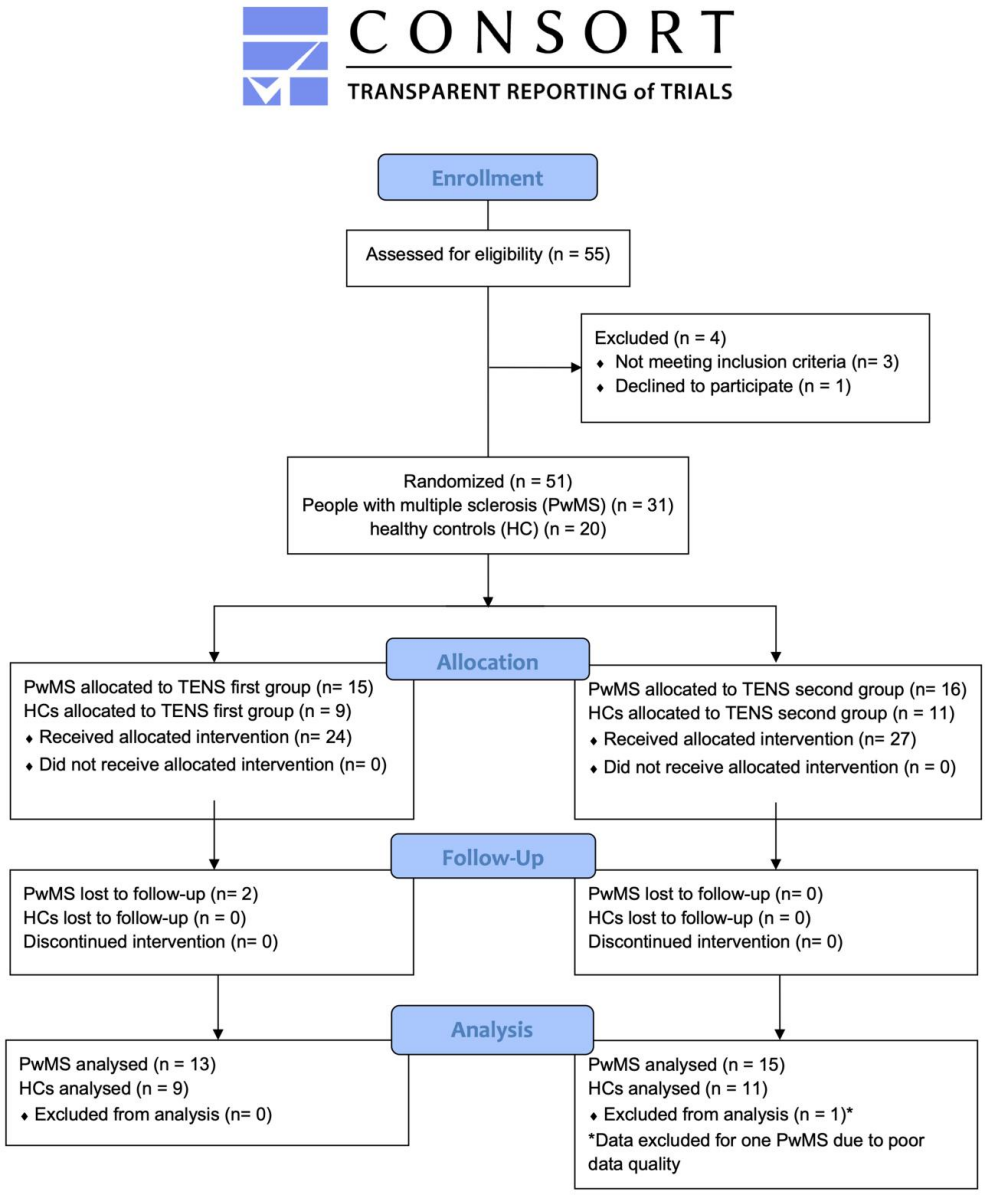
**Consort diagram.** Flowchart illustrating trial recruitment, randomization and retention of participants. A total of 55 participants were assessed for eligibility, with four excluded (three did not meet inclusion criteria, one declined to participate). The remaining 51 participants (31 people with multiple sclerosis (PwMS) and 20 healthy controls (HC)) were randomized into the two transcutaneous electrical nerve stimulation (TENS) conditions. During the trial, two PwMS were lost to follow-up and one had poor data quality, resulting in 28 PwMS and 20 HC participants in this analysis.

A total of 55 participants were screened and 51 participants (31 PwMS and 20 HCs) were enrolled. Of these, 29 PwMS and 20 HCs completed the study, with one PwMS excluded from analysis due to poor data quality (Fig. 2). PwMS and HCs did not differ in age, activity, body mass index, RPE during Late Adapt or baseline SLA. However, PwMS exhibited significantly higher fatigue, greater mCTSIB proprioceptive sway, reduced vibration perception threshold, reduced fast walk speeds and shorter baseline step lengths (Table 1). This sample of PwMS had relatively low impairment (EDSS = 3.2) and was highly active compared with average PwMS.^61^

**Table 1:** Participant characteristics

Characteristic	PwMS mean (SD)	HC mean (SD)	*P*
N	28	20	
Age	53.5 (10.7)	53.8 (14.0)	0.953
Sex (female/male)	19/9	12/8	0.760
Body mass index	24.9 (5.3)	25.4 (4.5)	0.746
Activity (hours per week)	5.1 (3.1)	5.5 (4.4)	0.735
Fallen in past 6 months (yes/no)	6/22	0/20	0.034*
History of sensory impairments (e.g. numbness, tingling) (yes/no)	24/4	0/20	<0.001***
Years since multiple sclerosis diagnosis^a^	12.8 (17.0)		
Expanded Disability Status Scale^a^	3.5 (1.5)		
12-Item Multiple Sclerosis Walking Scale^a^	16.5 (12.3)		
Modified Fatigue Impact Scale^a^	27.5 (16.8)	4.5 (9.3)	<0.001***
Beck Depression Inventory^a^	4 (7)	0 (3)	0.008**
Oral Symbol Digit Modalities Test	73.4 (13.6)	77.4 (15.7)	0.368
Vibration perception threshold^a^	5.5 (2.4)	6.4 (1.1)	0.016*
mCTSIB total sway (cm)^a^	205.0 (179.0)	161.5 (57.0)	0.063
mCTSIB proprioception sway (cm)^a^	34.5 (41.5)	25.5 (10.5)	0.005**
Rate of perceived exertion during Late Adapt^a^	3.6 (0.7)	3.4 (1)	0.325
Preferred walk speed (m/s)	1.16 (0.20)	1.19 (0.12)	0.454
Fast walk speed (m/s)	1.40 (0.24)	1.55 (0.16)	0.018*
Baseline step length (mm)	563.0 (68.5)	602.4 (55.4)	0.003**
Baseline step length asymmetry	0.021 (0.017)	0.016 (0.012)	0.089
Rectus femoris TENS amplitude (mA)^b^	13.7 (7.9)	11.4 (5.7)	0.103
Tibialis anterior TENS amplitude (mA)^b^	16.8 (8.9)	14.2 (5.7)	0.086

^a^Data were not normally distributed. Values are reported as median (IQR) and group differences were tested using the Mann–Whitney *U* test.

^b^Amplitudes were averaged across the left and right sides for each participant.

Participant characteristics and baseline clinical measures for people with multiple sclerosis (PwMS) and healthy control (HC) groups, presented as mean ± (standard deviation). *P*-values indicate group differences when applicable. mCTSIB, Modified Clinical Test of Sensory Interaction in Balance; TENS, Transcutaneous Electrical Nerve Stimulation. *P < 0.05; **P < 0.01; ***P < 0.001.

### SLA adaptation outcomes

Adaptation, or changes in SLA during split-belt walking, was quantified using the following four outcomes: adaptation magnitude, early change, after-effect and savings. Full results are summarized in Table 2.

**Table 2:** Step length asymmetry adaptation

Adaptation magnitude			
Significant predictor	*df*	*F*	*P*
Group	1, 44	5.7	0.021*
Visit	1, 44	22.9	<0.001***
TENS condition	1, 44	5.0	0.031*

Pairwise	EMM (SE)	*P*	adj-*P*	*d*
PwMS–HC	−0.022 (0.009)	0.021	0.042*	0.81
PwMS: Visit 2–Visit 1	−0.021 (0.007)	0.004	0.008**	0.80
HC: Visit 2–Visit 1	−0.031 (0.008)	<0.001	0.001**	1.18
PwMS: TENS ON–TENS OFF	−0.019 (0.007)	0.008	0.017*	0.73
HC: TENS ON–TENS OFF	−0.005 (0.008)	0.548	0.5483	0.19

Early change			
Significant predictor	*df*	*F*	*P*
Visit	1, 44	20.2	<0.001***

Pairwise	EMM (SE)	*P*	adj-*P*	*d*
PwMS: Visit 2–Visit 1	0.024 (0.007)	0.001	0.003**	0.92
HC: Visit 2–Visit 1	0.025 (0.008)	0.005	0.009**	0.95

After-effect			
Significant predictor	*df*	*F*	*P*
Visit	1, 44	20.4	<0.001***

Pairwise	EMM (SE)	*P*	adj-*P*	*d*
PwMS: Visit 2–Visit 1	0.012 (0.004)	0.002	N/A^a^	0.85
HC: Visit 2–Visit 1	0.014 (0.004)	0.002	N/A^a^	1.02

Savings^b^			
Significant predictor	*df*	*F*	*P*
Initial Adapt: TENS Condition	1, 44	12.7	<0.001***
Early Adapt: TENS Condition	1, 44	6.3	0.016*

Pairwise	EMM (SE)	*P*	adj-*P*	*d*
Initial Adapt, PwMS: TENS ON–TENS OFF	0.050 (0.014)	< 0.001	0.005**	1.35
Initial Adapt, HC: TENS ON–TENS OFF	0.022 (0.017)	0.199	0.442	0.59
Early Adapt, PwMS: TENS ON–TENS OFF	0.042 (0.014)	0.005	0.014*	1.13
Early Adapt, HC: TENS ON–TENS OFF	0.006 (0.017)	0.732	0.878	0.16

^a^Multiple comparisons corrections were not applied for after-effect, as it represents a single value across the entire paradigm, with no comparisons across different phases.

^b^Since savings is defined as the difference between Visit 1 and Visit 2 for Initial and Early Adapt, random effects were not included in the model, as only one value was calculated per participant.

Linear mixed-effects model results and subsequent pairwise comparisons using estimated marginal means (EMM; adjusted group means) and standard error (SE) for adaptation magnitude (A), early change (B), after-effect (C) and savings (D). All models considered group (people with multiple sclerosis (PwMS) versus healthy controls (HC)), visit (Visit 1 versus Visit 2) and transcutaneous electrical nerve stimulation (TENS) condition (TENS ON versus TENS OFF) as fixed effects and participant as a random effect. Only statistically significant main effects and their associated pairwise comparisons are reported. No significant interactions were present for any outcome. *P < 0.05; **P < 0.01; ***P < 0.001.

#### Adaptation magnitude

**Figure 3:**
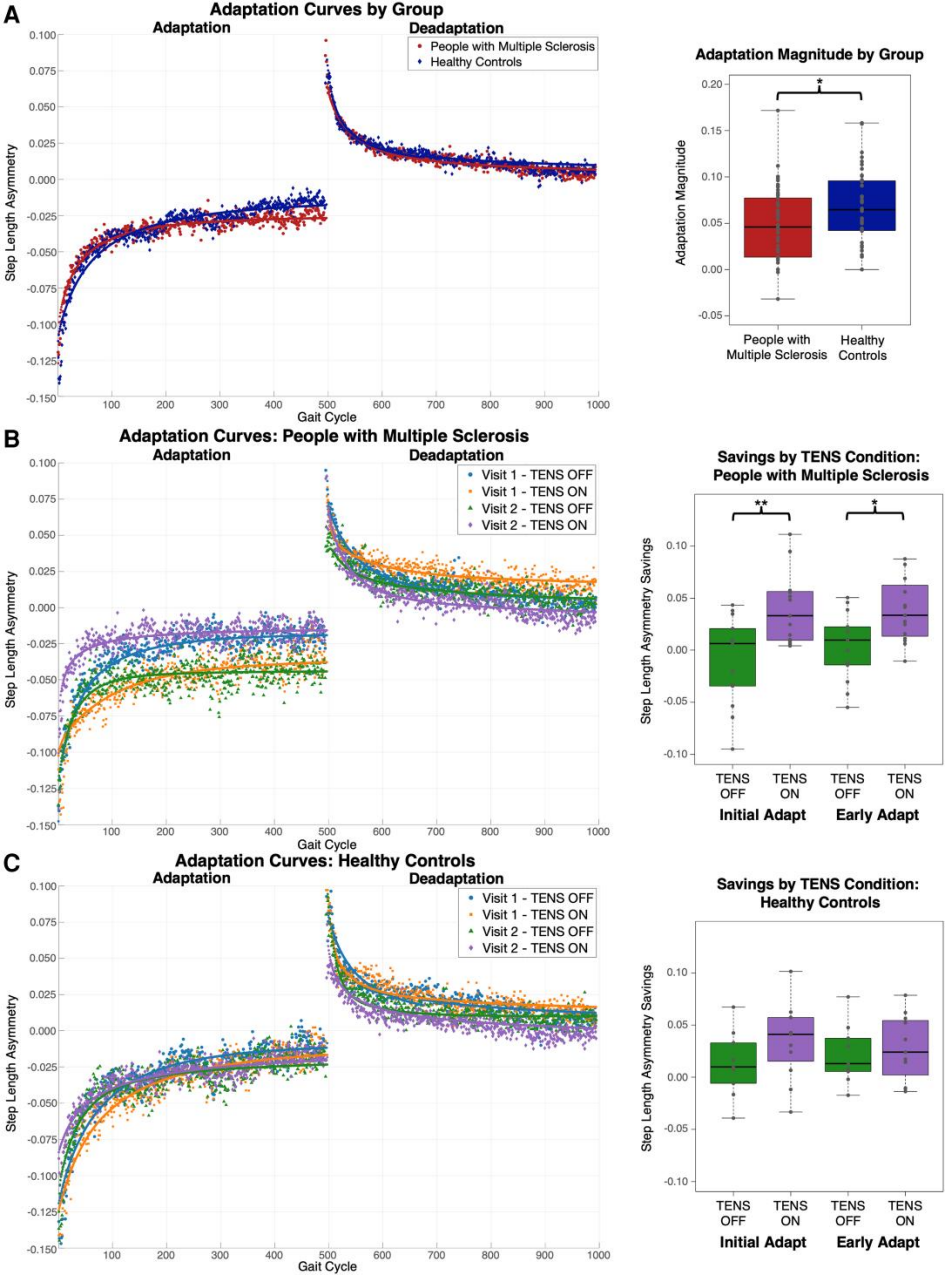
**Step length asymmetry (SLA) adaptation.** (**A**) SLA adaptation curves and boxplots for people with multiple sclerosis (PwMS) and healthy controls (HC) averaged across all visits and conditions. While early change remained similar, PwMS exhibited reduced adaptation magnitude compared with HCs (*t*(44) = −2.39, adj-*P* = 0.042, *d* = 0.81). (**B**) SLA adaptation curves and boxplots for PwMS for each visit and condition. Transcutaneous electrical nerve stimulation (TENS) did not significantly affect early change during adaptation; however, at Visit 2, TENS ON (purple) significantly enhanced adaptation savings at Initial Adapt (*t*(44) = 3.56, adj-*P* = 0.005, *d* = 1.35) and Early Adapt (*t*(44) = 2.98, adj-*P* = 0.014, *d* = 1.13) compared with TENS OFF (green). During deadaptation, PwMS showed reduced early change at Visit 2, while savings did not differ between TENS conditions. (**C**) SLA adaptation curves and boxplots for HC for each visit and condition. TENS had no significant impact on any adaptation variable. However, early change was greater at Visit 2 compared with Visit 1 during adaptation. Similar to PwMS, reduced early change was observed during deadaptation at Visit 2. Each data point in all adaptation curves (**A–C**) represents the group-average SLA for a single gait cycle (x-axis) with different marker symbols and colours indicating experimental conditions, totalling 1000 data points per condition. For visual purposes SLA curves were interpolated, but all analyses were based on the uninterpolated data. Linear mixed-effects model results of all adaptation outcomes are reported in Table 2.

Adaptation magnitude showed significant main effects of group, visit and TENS condition with no significant interactions (Table 2). Across all visits and TENS conditions, PwMS exhibited reduced adaptation magnitude compared with HCs (EMM = 0.022, adj-*P* = 0.042, *d* = 0.81) (Fig. 3A). As expected, PwMS and HCs had reduced adaptation magnitude at Visit 2. Importantly, with TENS ON, adaptation magnitude was reduced in PwMS (EMM = −0.019, adj-*P* = 0.017, *d* = 0.73), but not in HCs (EMM = −0.005, adj-*P* = 0.548, *d* = 0.19). In contrast, no significant main effects or group differences were observed for deadaptation magnitude.

#### Early change

For early change, visit was the only significant main effect with greater early change at Visit 2 compared with Visit 1, indicating a faster adaptation rate, for both PwMS (EMM = 0.024 adj-*P* = 0.003, *d* = 0.92) and HCs (EMM = 0.025, adj-*P* = 0.009, *d* = 0.95) (Table 2; Fig. 3B and C). When comparing early change at Visit 1 only, no significant difference was observed between TENS ON and TENS OFF conditions in PwMS (EMM = −0.002, *P* = 0.898, *d* = 0.09), despite its visual appearance. Additionally, during deadaptation PwMS and HCs showed reduced early change at Visit 2 compared with Visit 1 (PwMS: EMM = −0.011, adj-*P* = 0.023, *d* = 0.62; HC: EMM = −0.015, adj-*P* = 0.009, *d* = 0.86).

#### After-effect

After-effect also showed a significant main effect of visit and was not influenced by group or TENS condition (Table 2). For PwMS and HCs, after-effect decreased on Visit 2 compared with Visit 1 (PwMS: EMM = −0.012, *P* = 0.002, *d* = 0.85; HC: EMM = −0.014, *P* = 0.002, *d* = 1.02), which is consistent with previous reports.^53^

#### Savings

For savings, TENS condition was the only significant main effect at both Initial Adapt and Early Adapt (Table 2). Among PwMS, receiving TENS ON at Visit 2 resulted in greater savings compared with receiving TENS OFF (Initial Adapt: EMM = 0.050, adj-*P* = 0.005, *d* = 1.35; Early Adapt: EMM = 0.042, adj-*P* = 0.014, *d* = 1.13) (Fig. 3B). Conversely, for HCs with TENS ON at Visit 2, this effect was not significant (Fig. 3C). For deadaptation, TENS condition had no effect on savings at either Initial Deadapt or Early Deadapt for either group.

Given significantly greater savings with TENS ON at Visit 2, stepwise regression models predicting savings were performed across all participants, incorporating all demographic, clinical and functional variables included in Table 1. While TENS condition remained significant in both models, savings at Initial Adapt was predicted by vibration perception threshold (*t*(44) = 2.6, *P* = 0.013) and savings at Early Adapt was predicted by mCTSIB proprioceptive sway (*t*(42) = 2.3, *P* = 0.024) and vibration perception threshold marginally (*t*(42) = 2.0, *P* = 0.056).

### Channel-wide activation

**Figure 4:**
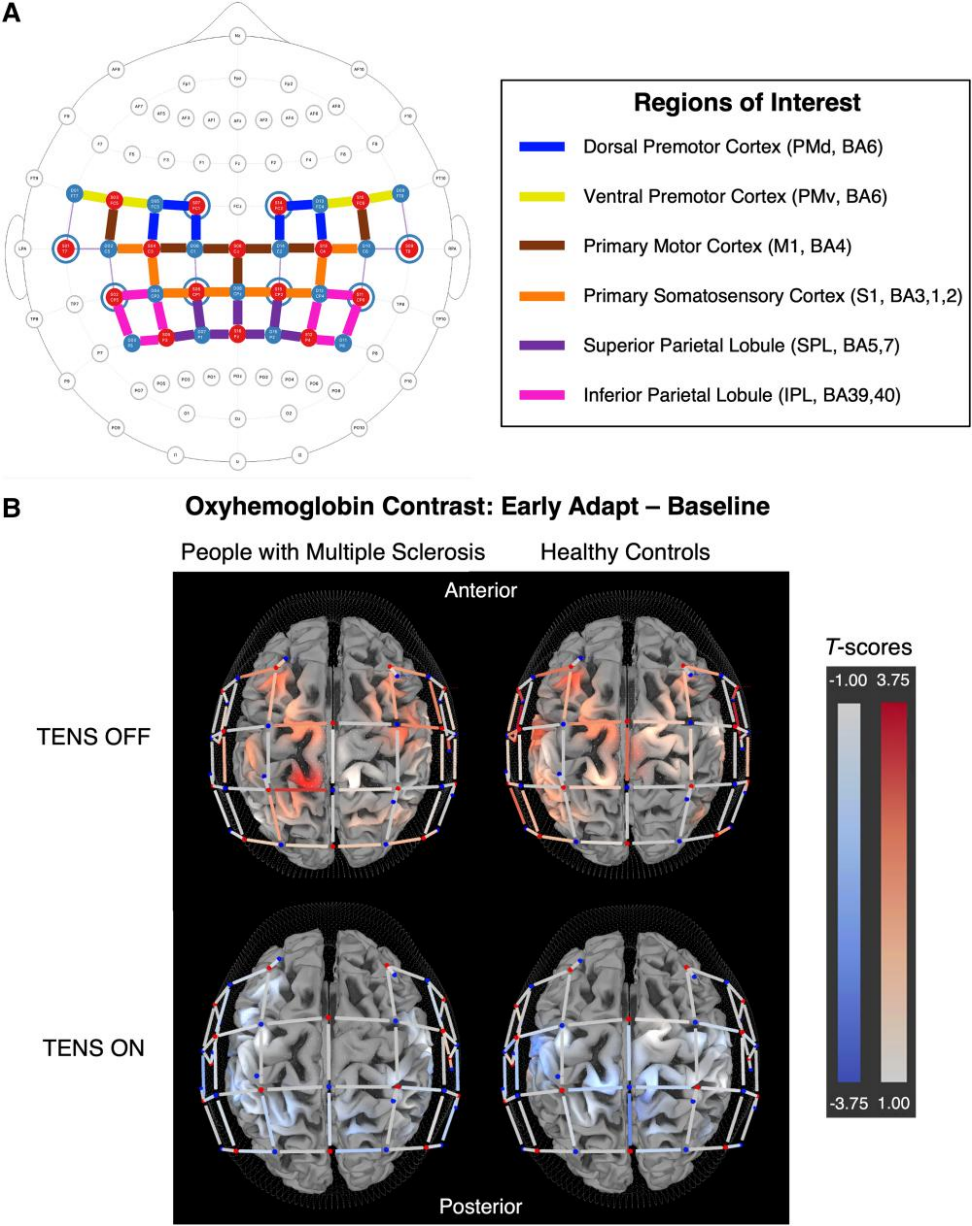
**Functional near-infrared spectroscopy (fNIRS) results.** (**A**) fNIRS channel locations are shown relative to the 10–10 system. Sources are represented by red circles, detectors by blue circles and short-distance detectors by blue rings. Each source-detector line indicates an individual channel, with the thick coloured lines highlighting channels assigned to a specific region of interest (ROI) based on their Brodmann areas (BA). ROIs included dorsal and ventral premotor areas (PMd, PMv), primary motor and somatosensory areas (M1, S1) and superior and inferior parietal lobules (SPL, IPL). (**B**) Cortical activation with transcutaneous electrical nerve stimulation (TENS) OFF and TENS ON. Group level *t*-score maps oxyhaemoglobin (HbO) beta change for the Early Adapt—Baseline contrast across premotor, sensorimotor and posterior parietal regions for people with multiple sclerosis (PwMS) (N = 28) and healthy controls (HC) (N = 20). With TENS OFF, both groups exhibited channel-wide increases in activation, including significant activation of the dorsal premotor cortex (PMd) during Early Adapt compared with Baseline. In contrast, with TENS ON, channel-wide activation was significantly reduced compared with TENS OFF for both groups, with no increase from Baseline to Early Adapt. Among only PwMS, this reduced activation with TENS ON was observed specifically in the PMd, primary motor (M1), primary sensory (S1) and superior parietal lobule (SPL) regions of interest (ROIs). Linear mixed-effects model results of all ROIs are reported in Table 3.

Across all channels, TENS condition had a significant main effect. Surprisingly, there were no effects of visit or group, nor any interactions (Table 3). When comparing timepoints, HbO beta at Early Adapt was significantly greater than Baseline with TENS OFF for PwMS (EMM = 0.054, *P* = 0.037, *d* = 0.56) and HCs (EMM = 0.073, *P* = 0.017, *d* = 0.76). Additionally, when comparing TENS conditions for the Early Adapt–Baseline contrast, TENS ON resulted in significantly reduced HbO beta compared with TENS OFF in both groups (PwMS: EMM = −0.078 *P* = 0.007, *d* = 0.76; HC: EMM = −0.089 *P* = 0.008, *d* = 0.88) (Fig. 4).

**Table 3:** Functional near-infrared spectroscopy (fNIRS) oxyhaemoglobin (HbO) beta changes

Channel-wide activation			
Predictor	*df*	*F*	*P*
Group	1, 44	0.3	0.579
Visit	1, 44	0.2	0.692
TENS Condition	1, 44	15.6	<0.001***

Contrast	EMM (SE)	*P*	*d*
PwMS: Early Adapt–Baseline	0.054 (0.026)	0.037*	0.56
HC: Early Adapt–Baseline	0.073 (0.031)	0.017*	0.76
PwMS: TENS ON–TENS OFF	−0.078 (0.027)	0.007**	0.76
HC: TENS ON–TENS OFF	−0.089 (0.032)	0.008**	0.88

ROIs: Early Adapt–Baseline Contrast					
Group	ROI	EMM (SE)	*P*	adj-*P*	*d*
PwMS	PMd	0.097 (0.034)	0.004	0.026*	0.77
HC	PMd	0.099 (0.040)	0.014	0.042*	0.78
PwMS	PMv	0.021 (0.043)	0.626	0.626	0.13
HC	PMv	0.085 (0.051)	0.093	0.186	0.54
PwMS	M1	0.064 (0.029)	0.030	0.090	0.58
HC	M1	0.088 (0.035)	0.012	0.042*	0.81
PwMS	S1	0.065 (0.034)	0.056	0.113	0.51
HC	S1	0.039 (0.040)	0.329	0.329	0.31
PwMS	SPL	0.055 (0.036)	0.124	0.187	0.41
HC	SPL	0.044 (0.042)	0.303	0.329	0.33
PwMS	IPL	0.037 (0.037)	0.316	0.380	0.27
HC	IPL	0.061 (0.043)	0.160	0.239	0.45

ROIs: TENS ON–TENS OFF Contrast					
Group	ROI	EMM (SE)	*P*	adj-*P*	*d*
PwMS	PMd	−0.123 (0.040)	0.003	0.019*	0.84
HC	PMd	−0.090 (0.047)	0.060	0.090	0.61
PwMS	PMv	−0.007 (0.049)	0.888	0.888	0.04
HC	PMv	−0.061 (0.59)	0.301	0.301	0.33
PwMS	M1	−0.085 (0.038)	0.029	0.043*	0.61
HC	M1	−0.105 (0.045)	0.023	0.069	0.75
PwMS	S1	−0.106 (0.038)	0.008	0.024*	0.75
HC	S1	−0.113 (0.045)	0.017	0.069	0.79
PwMS	SPL	−0.104 (0.040)	0.013	0.026*	0.70
HC	SPL	−0.094 (0.047)	0.054	0.090	0.63
PwMS	IPL	−0.059 (0.045)	0.197	0.237	0.35
HC	IPL	−0.088 (0.054)	0.110	0.131	0.52

Linear mixed-effects model results and estimated marginal means (EMM) of channel-wide and region of interest (ROI) oxyhaemoglobin (HbO) beta changes for people with multiple sclerosis (PwMS) and healthy controls (HC). Contrasts include the difference between Early Adaptation and Baseline, as well as TENS ON–TENS OFF for the Early Adaptation-Baseline contrast. ROIs included dorsal and ventral premotor areas (PMd, PMv), primary motor and somatosensory cortices (M1, S1) and superior and inferior parietal lobules (SPL, IPL). Individual ROI ANOVA results available in Supplementary Table 2. *P < 0.05; **P < 0.01; ***P < 0.001.

### ROI-level activation

Linear mixed-effects model results of individual ROIs are shown in Supplementary Table 2. A similar trend of increased HbO beta during Early Adapt compared with Baseline with TENS OFF was apparent at the ROI level. For PwMS, a large increase in HbO beta at Early Adapt occurred exclusively in PMd (EMM = 0.097, adj-*P* = 0.026, *d* = 0.77), while M1 and S1 showed moderate effects that did not survive corrections. For HCs, HbO beta in PMd (EMM = 0.099, adj-*P* = 0.042, *d* = 0.78) and M1 (EMM = 0.088, adj-*P* = 0.042, *d* = 0.81) was increased at Early Adapt (Table 3).

When comparing TENS OFF with TENS ON conditions across both visits, PwMS exhibited a significant large-effect decrease in HbO beta with TENS ON in PMd (EMM = −0.123, adj-*P* = 0.019, *d* = 0.84), and significant moderate-effect decreases in M1, S1 and SPL. For HCs, no comparisons survived corrections for decreased HbO beta with TENS ON, but moderate effects were observed in PMd, M1, S1 and SPL (Table 3).

To evaluate whether demographic, clinical and functional variables listed in Table 1 accounted for variance in cortical activation, stepwise regression models were performed for each ROI. TENS condition remained significant in all ROIs. PMd HbO beta was additionally predicted by vibration perception threshold (*t*(87) = −2.0, *P* = 0.048) and body mass index (*t*(87) = −2.6, *P* = 0.010).

### Relationship between savings and cortical activation

**Figure 5:**
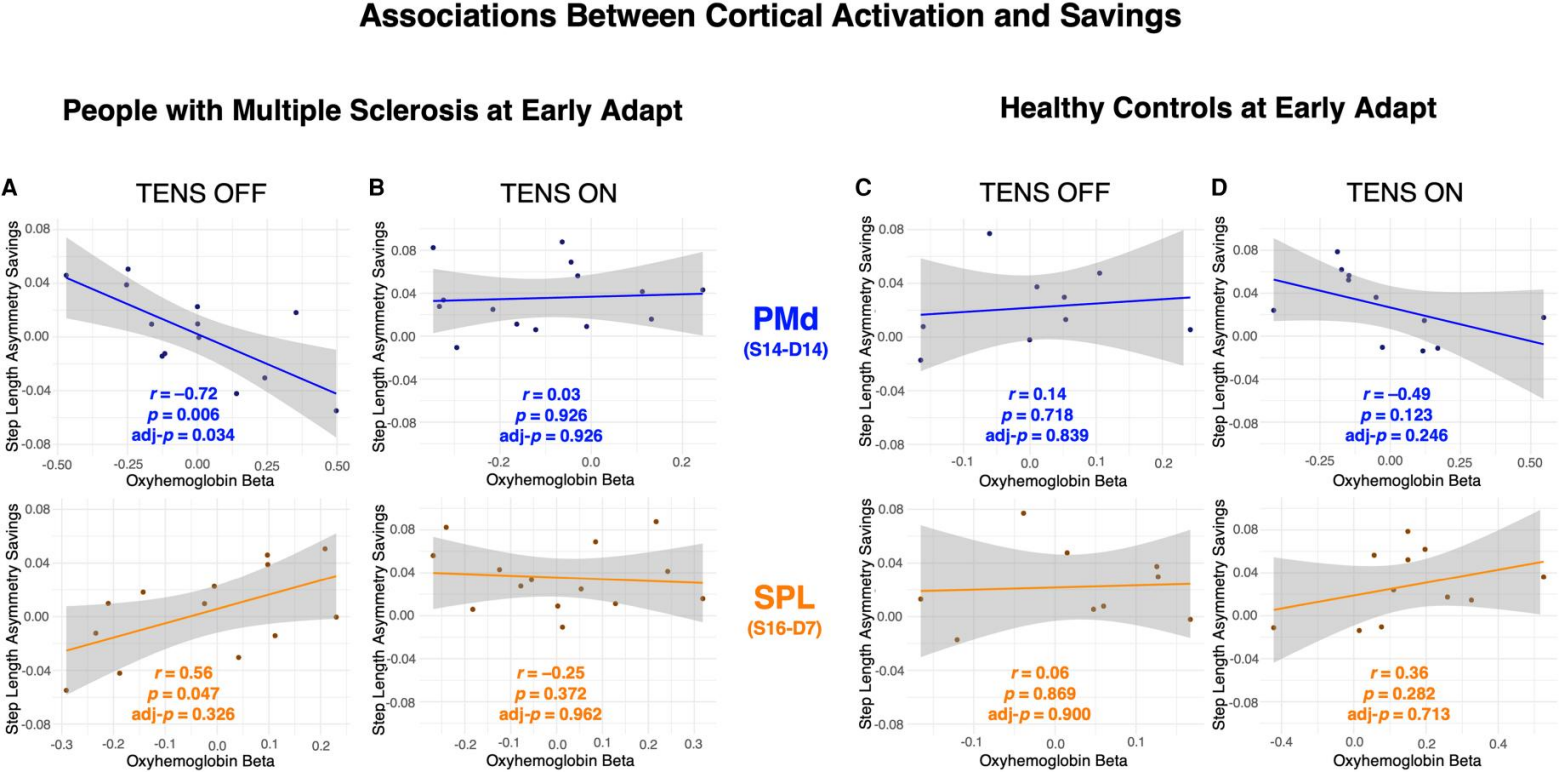
**Associations between cortical activation and savings.** Correlation plots showing relationships between step length asymmetry savings and cortical activation, quantified as oxyhaemoglobin (HbO) beta, at Early Adapt. (**A**) In people with multiple sclerosis (PwMS), a strong negative correlation was observed between savings and HbO beta in a dorsal premotor cortex (PMd) channel, with a potential correlation in a superior parietal lobule (SPL) channel (N = 13). (**B**) With transcutaneous electrical nerve stimulation (TENS) ON, these correlations were no longer present in PwMS (N = 15). In healthy controls (HCs), no correlations were observed at these channels under either (**C**) TENS OFF (N = 9) or (**D**) TENS ON (N = 11) conditions. Pearson correlations were used for all associations shown. Each data point represents an individual participant's average step length asymmetry savings and corresponding HbO beta in the specified channel.

In line with our hypothesis that TENS modulates activation in sensorimotor regions and the finding of significantly greater savings with TENS, correlations between HbO beta and savings at Early Adapt were examined. While no significant correlations were observed at the ROI level, a trending relationship in PwMS at PMd (*r* = −0.63, adj-*P* = 0.119) was insignificant after correction, prompting an exploratory analysis of channel-specific correlations within the hypothesized regions of PMd, PMv, SPL and IPL. In PwMS with TENS OFF, a strong correlation was observed in a PMd channel (S14-D14: *r* = *−*0.72, adj-*P* = 0.034) and a potential correlation in a SPL channel (S16-D17: *r* = 0.56, adj-*P* = 0.326) that lacked significance after correction. Interestingly, these correlations were absent for PwMS with TENS ON and for HCs in both TENS conditions suggesting that individuals who required greater recruitment of PMd during adaptation tended to show poorer savings across visits (Fig. 5).

To examine whether broader cortical activation patterns predicted savings, we computed a stepwise linear regression model including HbO beta from all ROIs. In PwMS with TENS OFF, activation in PMd (*t(8)* = −4.5, *P* = 0.001) and SPL (*t(8)* = 2.9, *P* = 0.016) contributed significantly to the model, which explained a substantial proportion of variance in savings (*R^2^* = 0.68, adj-*R*^2^ = 0.61, *F*(2,10) = 10.4, *P* = 0.004) further supporting the relationship between activation in these regions and savings in PwMS. No meaningful model contributions were observed in PwMS with TENS ON (*R^2^* = 0.32, adj-*R*^2^ = 0.14, *F*(3,11) = 1.7, *P* = 0.219).

## Discussion

This randomized controlled trial investigated the impact of TENS on locomotor adaptation in PwMS and HCs. Contrary to our hypothesis, TENS did not improve adaptation rate. Rather, TENS significantly enhanced adaptation savings in PwMS, as reflected by faster relearning from Visit 1 to Visit 2, suggesting that TENS may not influence the immediate adaptation process but instead enhances retention or retrieval of previous learning. Additionally, TENS reduced cortical activation in both groups, particularly in PMd, which may indicate a shift towards more automatic control and reduced cortical involvement during adaptation.

### TENS enhances locomotor adaptation savings in PwMS

Contrary to our hypothesis, TENS did not impact early change, which reflects adaptation rate. Instead, savings were significantly greater at Initial Adapt and Early Adapt for PwMS who received TENS ON during Visit 2, compared with those who received TENS OFF. Relatedly, adaptation magnitude was decreased with TENS ON as increased savings caused the SLA adaptation curve to begin at a higher point, inherently limiting the extent of further adaptation. This suggests that TENS may not improve the initial learning rate but facilitates the recall of prior learning and accelerates relearning. One possible explanation for the lack of effect on early change is that PwMS had similar early change compared with HCs adaptation, leaving limited room for further improvement. Alternatively, TENS may primarily act on the retrieval processes rather than modulating online learning. Previous work from our group corroborates this finding. Whittier *et al*.^28^ found the greatest improvement across visits in HCs who received TENS ON during Visit 2 during a visuomotor stepping task. For HCs in our study, a similar pattern of improved savings with TENS ON during Visit 2 was observed with a moderate effect size (*d* = 0.59), though not significant. This suggests that while TENS could impact savings in HCs, its effects are more pronounced in PwMS. This disparity may reflect a ceiling effect in HCs, limiting the observable benefits of TENS for those without clinical sensory impairment.

Despite a clear effect of visit on after-effect,^52^ TENS condition did not influence after-effect. Sensorimotor adaptation relies on a two-rate process: an initial, rapidly flexible mechanism followed by a gradual, implicit recalibration of the forward model or the neural process that predicts sensory consequences from motor commands.^62-64^ Forward model recalibration, driven by sensory prediction errors (i.e. mismatches between expected and actual sensory feedback), is considered the primary mechanism underlying after-effects upon exposure to a different environment.^65-67^ Since the present data show enhanced savings at Initial Adapt and Early Adapt with TENS ON, yet no changes in after-effect, TENS is likely modulating the rapidly flexible mechanism, rather than recalibration. This may indicate TENS is more effective when sensory prediction errors are large and when recalling a previously established forward model.^68^ TENS is suggested to reduce sensorimotor uncertainty for state estimates,^28^ which may be particularly beneficial when large sensory prediction errors occur^37,68^ and in turn promote more precise error adjustments which are used to refine the established forward model. Mechanistically, prior work suggests applying TENS primes ascending Aα and Aβ sensory fibres potentially by altering membrane properties that enhance excitability and thus firing rate.^20,69-71^ This effect would increase the quantity of sensory information transmitted from the periphery to the CNS.^32,34,72^ Separately, TENS, can enhance the detection of weak sensory signals through stochastic resonance, in which the presence of low level electrical noise enhances signal processing in the nervous system.^73-75^ This improved signal detection likely supports better sensorimotor integration at cortical and cerebellar levels, facilitating more accurate state estimates and sensory predication errors. In addition to TENS condition, greater vibration perception threshold and mCTSIB proprioceptive sway were predictive of greater savings, emphasizing the importance of sensory and proprioceptive input in generating adaptation savings, which is line with other motor learning findings.^11,76-79^ Prior work has also shown that TENS can reduce sway and improve proprioception, further highlighting the relevance of TENS for improving these inputs.^20,25,71,80^

Notably, the Visit 1-TENS OFF and Visit 2-TENS ON curves reflect performance from the same individuals, as do the Visit 1-TENS OFF and Visit 2-TENS ON curves. This within-participant consistency likely contributes to the visual similarity between the adaptation profiles across visits in PwMS, particularly at Late Adapt (Fig. 3B). However, there were no differences between TENS OFF and TENS ON conditions at Late Adapt for Visit 1 (EMM = −0.019, adj-*P* = 0.282, *d* = 1.00) or Visit 2 (EMM = 0.021, adj-*P* = 0.225, *d* = 1.12), but large effects at both visits may suggest some highly varying, non-significant differences that reflect individual variability rather than an effect of TENS. Importantly, these curves were baseline-adjusted and demographic factors were consistent across groups, minimizing the likelihood that these observed differences stem from overt sample disparities. By design, our analysis compares savings within participants, ensuring the improvements with TENS ON during Visit 2 are not influenced by sample heterogeneity.

### TENS reduces cortical activation

PwMS and HCs exhibited greater cortical activation, represented by increased HbO beta, during Early Adapt compared with Baseline. This increase was significant at the channel-wide level and at the ROI level for PMd in both groups and M1 for HCs only. Comparing activation between TENS conditions, TENS ON resulted in less cortical activation during the Early Adapt–Baseline contrast, which quantified the adaptation-related activation independent of treadmill walking. Unlike the savings findings, this effect was consistent across visits and groups, with both PwMS and HCs exhibiting decreased activation with TENS ON for both visits. Somewhat surprisingly, PwMS did not have more activation than HCs; however, this study did not measure activation in the prefrontal cortex, a primary area where increased activation is commonly observed during motor tasks in PwMS.^19,42^ This effect of TENS was strongest at the channel-wide level but also demonstrated significant differences with moderate to large effect sizes at the ROI level. Among the ROIs, PMd, M1, S1 and SPL exhibited significant decreases in activation with TENS ON for PwMS but not HCs. PMd is particularly relevant for sensorimotor adaptation as it is highly influential in motor planning and a primary node in the cortico-cerebellar loop.^81-83^ During feedforward control, sensory prediction errors processed by the cerebellum relay through the ventrolateral thalamus to PMd, M1, S1 and SPL where they contribute to refining motor plans, integrating sensory information and updating forward models of limb position and movement.^82-84^ The extent of premotor activation during this process depends on the magnitude of sensory prediction errors and the complexity of motor planning required to update subsequent motor output.^83^ In this study, greater PMd activation was additionally predicted by worse vibration perception threshold, emphasizing the impact of sensory function on cortical activation.

Similar to its effect on improved savings, TENS likely reduces cortical activation by enhancing firing rate of Aα and Aβ sensory fibres. These fibres are preferentially activated due to the longer pulse widths used in TENS, which selectively target large-diameter afferents without inducing muscle contractions.^20,31,70,85^ By reducing sensorimotor uncertainty and enhancing input, TENS may facilitate more efficient integration of sensory prediction errors within the cortico-cerebellar loop, thereby reducing the reliance on cortical regions, such as PMd, for complex motor planning. This observed reduction in cortical activation suggests a shift away from higher-order motor control, promoting greater automaticity during adaptation. With less cortical involvement, the reliance on subcortical structures, particularly the cerebellum likely increases. Notably, increased cerebellar engagement, as evidenced by reduced cerebellar-brain inhibition, has been associated with greater locomotor adaptation,^17^ suggesting that strategies relying more on cerebellar-driven control are likely less demanding and more effective for sensorimotor adaptation.^12^

While no previous studies have investigated the effect of TENS on functional cortical activation, other work has demonstrated cortical changes with the application of similar sensory stimulation using other imaging modalities.^34^ Using transcranial magnetic stimulation, Celnik *et al*.^33^ identified that sensory stimulation improved dexterity and reduced GABAergic intracortical inhibition, which is also linked with increased cortical representation.^86^ Interestingly, one group found that cortical motor representations, were increased after 3 weeks of daily TENS in HCs^35^ but in a separate study found decreased representation in PwMS.^87^ Additionally, EEG data have reported that high-frequency vibration shortened task completion time and decreased beta power over the sensorimotor cortex in people with Parkinson's disease, indicating enhanced automaticity of the task.^88^ Other work has also suggested that TENS reduces sensorimotor excitability, reflected by increased alpha-2 power, during a single-leg balance task.^89^ Conversely, while one study identified improved motor skill acquisition as a result of sensory stimulation, this was associated with increased N30 amplitudes in the sensorimotor areas.^29^ However, this task involved sequence learning, which is less cerebellar-driven and more reliant on cortical processes compared with sensorimotor adaptation.^67^ Overall, while our findings and others’ suggest TENS may improve automaticity, further research is needed to uncover the distinct neural changes that occur due to TENS.

### Cortical activation predicts savings in PwMS with TENS OFF only

Greater HbO beta in a PMd channel was associated with reduced savings, highlighting that increased PMd activation, reflecting higher-order motor involvement, may impede the efficiency of relearning during sensorimotor adaptation. Interestingly, this strong association was present only in PwMS with TENS OFF. With TENS ON this correlation was absent in PwMS, suggesting that adaptation is less influenced by PMd activation with TENS, potentially reflecting a shift towards more automatic or cerebellar-mediated control. In contrast, HCs exhibited no associations between activation and savings, indicating motor control strategies in PwMS more closely resemble those of HCs when TENS is applied. These correlations were further supported through regression models. In a model including all ROIs, PMd and SPL, explained a substantial portion of the variance in savings across participants only in PwMS with TENS OFF. These models were not significant in PwMS with TENS ON, reinforcing the idea that TENS may alter the neural contributions underlying savings in PwMS. While this pattern suggests that PwMS may rely on compensatory PMd engagement with TENS OFF, these analyses were exploratory, not corrected for all possible comparisons across subgroups, and the study was not powered for subgroup analyses. Increased PMd activation could reflect heightened cognitive or attentional demands in those with poorer adaptation or maladaptive recruitment of motor planning circuits that interfere with recalibration. Future studies using imaging methods such as fMRI are needed to determine whether TENS reduces reliance on compensatory cortical mechanisms and enhances cerebellar engagement or connectivity during adaptation.

### Limitations

One primary limitation of this study was the absence of a group that received TENS OFF or TENS ON across both visits. These groups would have been particularly relevant, as savings from Visit 1 to Visit 2 was the primary behavioural outcome influenced by TENS. Without these reference groups, it is possible that TENS ON during Visit 1 may have impaired savings at Visit 2, rather than TENS ON during Visit 2 improving savings as suggested throughout this article. However, Visit 1–TENS OFF was not significantly different than Visit 1–TENS ON for early change, late adaptation, adaptation magnitude or after-effect in PwMS or HCs, limiting the likelihood of this alternative hypothesis. Another design limitation is the absence of a sham TENS condition. This decision was intentional, as most sham protocols involve an initial stimulation period before ramping down, which would likely affect the initial and early adaptation phases that were central to our outcome measures. Importantly, because locomotor adaptation is an implicit process with minimal volitional control, the influence of placebo effects is likely limited. Additionally, while the acquired sample size aligns with the *a priori* power analysis and with similar studies,^43^ the counterbalancing between groups was imperfect due to participant dropout during the study, which affected randomization. Furthermore, achieving equal group sizes between PwMS and HCs could have enhanced statistical power. Another sampling limitation is that compared with other cohorts of PwMS with a similar number of years since diagnosis, this sample had lower disability scores and was quite active. Accordingly, split-belt treadmill walking is a relatively complex motor task that excludes many PwMS with higher disability, limiting the generalizability of these results. Additionally, while fNIRS measures neural activation during ecologically valid tasks such as walking, it is limited by its lower spatial resolution and depth compared with other neuroimaging methods like fMRI, which is especially important for ROI analyses. Furthermore, fNIRS can only measure cortical surface activation and does not capture subcortical or cerebellar contributions, which are essential for adaptation. Importantly, each ROI included multiple channels that were determined by their highest BA specificity. Averaging across multiple anatomically constrained channels helps reduce misclassification and improves the reliability of ROI-level interpretations. However, our conclusions regarding specific cortical regions should be interpreted as reflecting surface-level activity within these general anatomical boundaries, rather than precise localization.

### Clinical implications and future directions

While previous studies have demonstrated improvements in motor control with TENS, this is the first to emphasize the importance of treatment timing during motor learning. Specifically, TENS appears to enhance retention of motor learning in populations with sensory dysfunction and may be most effective when applied after initial skill acquisition, rather than during early learning. Consequently, clinicians may consider strategically introducing TENS in later rehabilitation sessions to reinforce previously learning skills and support motor automaticity, though the optimal timing of stimulation may depend on the specific task.^90^ This approach may help transition patients from conscious, effortful control to more automatic strategies. This shift is particularly important for functional movements, like walking or grasping, allowing patients to regain independence and reduce dual-task impairments. While this study applied TENS continuously during motor learning, future work should examine the optimal stimulation duration, intensity and potential impact of nerve accommodation during extended clinical use. Moreover, TENS may offer benefits for other rehabilitation strategies in neurological conditions by reducing reliance on compensatory neural recruitment and facilitating more natural motor control strategies.

Further research is needed to better understand the mechanisms of TENS-induced improvements in motor control, especially during motor learning. Investigating how TENS affects perceptual components of adaptation or proprioceptive testing would further elucidate its effects on the sensory system.^52,91^ To assess its generalizability, future studies should investigate the effect of TENS on other sensorimotor adaptations, including reaching and visuomotor perturbations, along with different motor learning modalities, like reinforcement learning.^67^ As a next step, the impact of TENS on overground transfer of locomotor adaptation should be explored. Recent evidence suggests that gradual adaptation improves overground transfer,^92^ likely due to modifying credit assignment of stepping errors.^43^ By decreasing sensorimotor uncertainty, TENS may augment credit assignment during adaptation, or potentially only be beneficial during the transfer stage of the paradigm, providing further insights into which stages of learning are the most responsive to TENS.

## Conclusion

This study is the first to demonstrate that TENS enhances sensorimotor adaptation in PwMS, highlighting the importance of treatment timing on savings. Specifically in PwMS with mild to moderate disability (median EDSS = 3.5), TENS did not affect adaptation rate during initial exposure to the split-belt treadmill but did enhance adaptation savings. This indicates that TENS may be particularly effective in enhancing the recall of prior learning or motor memories, resulting in faster relearning. The reduction in cortical activation in both groups, suggests a shift towards reduced cortical reliance and greater automaticity during learning, even without behavioural change in HCs. These findings indicate that TENS may have broader utility in populations with sensory impairments to amplify the retention of motor learning and promote greater automaticity, factors that are key for regaining functional independence during motor rehabilitation.

## Supplementary Material

fcaf255_Supplementary_Data

## Data Availability

The data that support the findings of this study are openly available in the Open Science Framework at https://osf.io/pgv37/. The code used for the analyses in this study is available on GitHub at https://github.com/andyhagen11/Transcutaneous-electrical-nerve-stimulation-enhances-locomotor-adaptation-savings-in-MS.
