# Supplementary material for: Transcutaneous electrical nerve stimulation enhances locomotor adaptation savings in people with multiple sclerosis
Source: Brain Commun. 2025 Jun 25;7(4):fcaf255. doi: 10.1093/braincomms/fcaf255 (PMC12238753; doi:10.1093/braincomms/fcaf255)
Supplement: fcaf255_Supplementary_Data [file fcaf255_supplementary_data.pdf]

Supplementary Table 1. Channel Locations and Region of Interest (ROI) Designations

Channel	Source	Detector	Channel coordinates (MNI)	Brodmann areas	ROI Designation
1	1	1	(-63,-9,-12)	21	
2	1	2	(-65,-18,4)	22,21	
3	2	2	(-63,-32,23)	22	
4	2	3	(-57,-57,21)	39	IPL
5	2	4	(-57,-48,38)	40,39	IPL
6	3	1	(-59,11,9)	6,44	PMv
7	3	2	(-62,-3,23)	4,43	M1
8	3	5	(-55,12,34)	6,44	PMv
9	4	2	(-60,-18,37)	3,2	S1
10	4	4	(-52,-34,52)	2,3,1	S1
11	4	5	(-50,-3,50)	6	PMd
12	4	6	(-42,-20,62)	4	M1
13	5	3	(-46,-72,30)	39	IPL
14	5	4	(-46,-61,46)	39,40	IPL
15	5	7	(-32,-73,47)	7	SPL
16	6	4	(-39,-48,60)	2,3,40	S1
17	6	6	(-27,-36,71)	4,3	
18	6	7	(-24,-62,62)	7	SPL
19	6	8	(-16,-50,72)	1,5	S1
20	7	5	(-38,12,55)	6,9	PMd
21	7	6	(-26,-5,68)	6	PMd
22	8	6	(-17,-20,74)	4,6	M1
23	8	8	(1,-35,75)	4	M1
24	8	14	(17,-21,75)	4,6	M1
25	9	9	(66,-8,-12)	21	
26	9	10	(67,-19,4)	22,21	
27	10	9	(61,11,8)	6,44	PMv
28	10	10	(64,-5,22)	4,43	S1
29	10	13	(56,12,33)	6,44	PMv
30	11	10	(65,-33,23)	22	
31	11	11	(58,-58,22)	39	IPL
32	11	12	(58,-48,38)	40,39	IPL
33	12	11	(47,-72,30)	39	IPL
34	12	12	(46,-62,47)	39,40	IPL
35	12	15	(33,-74,48)	7	SPL
36	13	10	(62,-20,37)	2,3,1	S1
37	13	12	(53,-35,52)	2,3,1	S1

38	13	13	(52,-4,48)	6	PMd
39	13	14	(42,-21,62)	4	M1
40	14	13	(39,12,54)	6,9	PMd
41	14	14	(27,-4,68)	6	PMd
42	15	8	(17,-50,73)	3,1,5	S1
43	15	12	(39,-49,60)	2,3,40	S1
44	15	14	(28,-36,71)	4,3	
45	15	15	(25,-62,63)	7	SPL
46	16	7	(-13,-73,56)	7	SPL
47	16	8	(2,-61,66)	5,7	SPL
48	16	15	(15,-73,57)	7	SPL

Channels formed by source-detector pairs were localized using Montreal Neurological Institute (MNI) coordinates and anatomical landmarks, identified through the Brodmann atlas. ROIs were determined based on the Brodmann area with the highest specificity for each channel. ROIs included dorsal and ventral premotor areas (PMd, PMv), primary motor and somatosensory cortices (M1, S1), and superior and inferior parietal lobules (SPL, IPL).

Supplementary Table 2. Region of Interest (ROI) Model Results

PMd Activation

Predictor	*df*	*F*	*P*	FDR-adj. *P*
Group	1, 44	0.3	0.595	0.838
Visit	1, 44	0.3	0.614	0.737
TENS Condition	1, 44	12.1	0.001	0.003**

PMv Activation

Predictor	*df*	*F*	*P*	FDR-adj. *P*
Group	1, 44	0.5	0.484	0.838
Visit	1, 44	0.3	0.577	0.737
TENS Condition	1, 44	0.8	0.378	0.378
Group:Visit Interaction	1,44	7.7	0.008	0.049*

M1 Activation

Predictor	*df*	*F*	*P*	FDR-adj. *P*
Group	1, 44	0.2	0.627	0.838
Visit	1, 44	0.5	0.491	0.737
TENS Condition	1, 44	10.6	0.002	0.003**

S1 Activation

Predictor	*df*	*F*	*P*	FDR-adj. *P*
Group	1, 44	0.8	0.366	0.838
Visit	1, 44	0.8	0.383	0.737
TENS Condition	1, 44	13.7	< 0.001	0.003**

SPL Activation

Predictor	*df*	*F*	*P*	FDR-adj. *P*
Group	1, 44	0.0	0.838	0.838
Visit	1, 44	0.0	0.968	0.968
TENS Condition	1, 44	10.2	0.002	0.003**

IPL Activation

Predictor	*df*	*F*	*P*	FDR-adj. *P*
Group	1, 44	0.0	0.772	0.838
Visit	1, 44	1.1	0.307	0.737
TENS Condition	1, 44	4.4	0.039	0.047*

Fixed-effects ANOVA results from linear mixed-effects models of oxyhemoglobin (HbO) change across individual regions of interest (ROIs). Surprisingly, only one significant interaction was present. ROIs included dorsal and ventral premotor areas (PMd, PMv), primary motor and somatosensory cortices (M1, S1), and superior and inferior parietal lobules (SPL, IPL). FDR = false discovery rate.

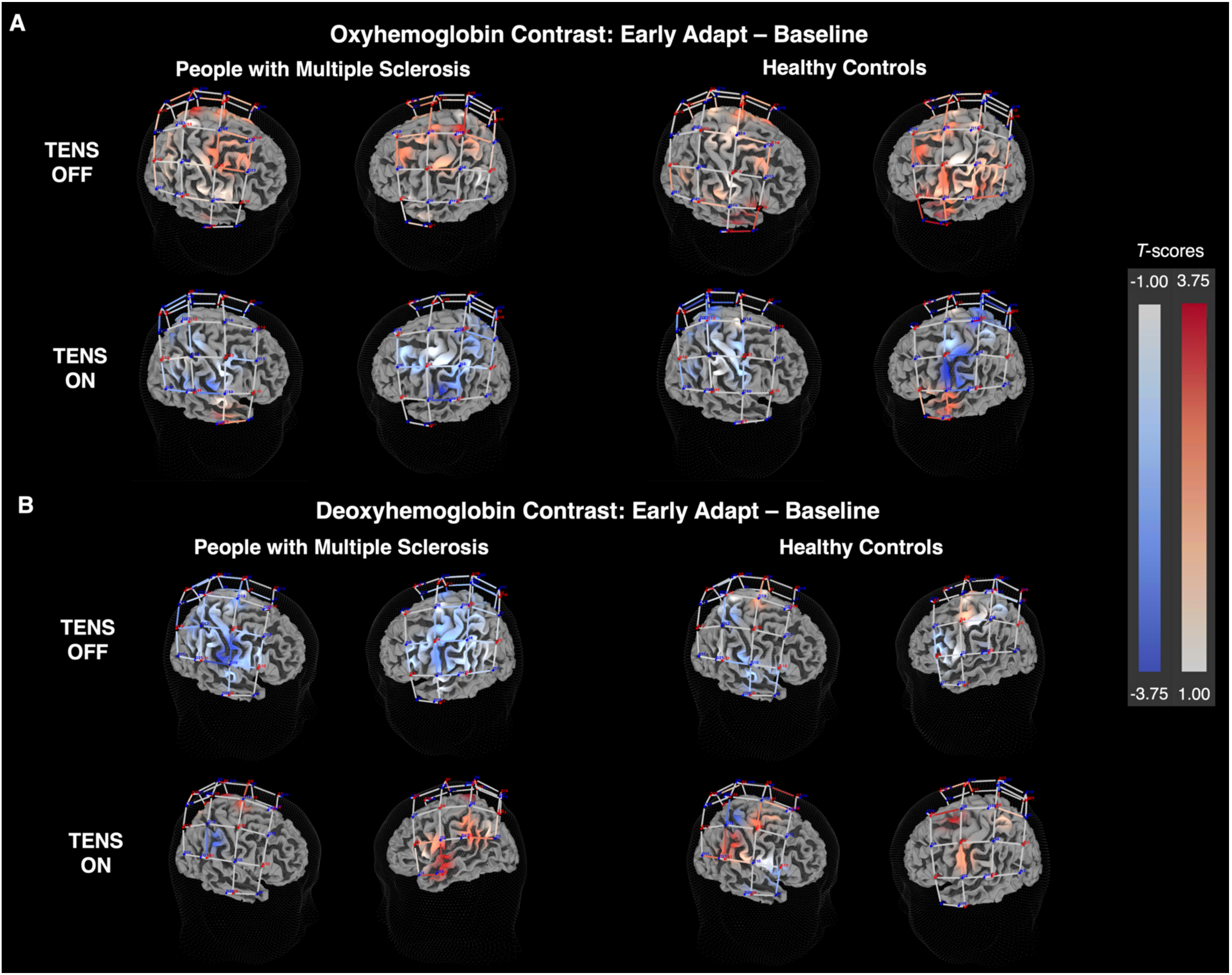

Supplementary Figure 1. Oxyhemoglobin (HbO) and deoxyhemoglobin (HbR) changes from Baseline to Early Adapt. **(A)** Bilateral views and *t*-score map showing HbO beta changes for the Early Adapt – Baseline contrast across groups and TENS conditions. **(B)** Bilateral views and *t*-score map of HbR beta changes for the Early Adapt – Baseline contrast across groups and TENS conditions. *T*-scores were calculated using a random effects model including 28 people with multiple sclerosis and 20 healthy controls.
